# ProsCan for Couples: Randomised controlled trial of a couples-based sexuality intervention for men with localised prostate cancer who receive radical prostatectomy

**DOI:** 10.1186/1471-2407-8-226

**Published:** 2008-08-08

**Authors:** Suzanne K Chambers, Leslie Schover, Kim Halford, Samantha Clutton, Megan Ferguson, Louisa Gordon, RA Gardiner, Stefano Occhipinti, Jeff Dunn

**Affiliations:** 1 Viertel Centre for Research in Cancer Control, Cancer Council Queensland, Brisbane, Australia; 2 School of Psychology, Griffith University, Brisbane, Australia; 3 National Executive, Australian Prostate Cancer Collaboration, Melbourne, Australia; 4 Department of Behavioral Science, University of Texas M.D. Anderson Cancer Center, Houston, USA; 5 Cancer Counselling Service, Cancer Council Queensland, Brisbane, Australia; 6 Cancer and Population Health Studies, Queensland Institute of Medical Research, Brisbane, Australia; 7 Department of Surgery, University of Queensland, Brisbane, Australia; 8 School of Social Science, University of Queensland, Brisbane, Australia; 9 School of Public Health, James Cook University, Townsville, Australia

## Abstract

**Background:**

Prostate cancer is the most common male cancer in the Western world. The most substantial long term morbidity from this cancer is sexual dysfunction with consequent adverse changes in couple and intimate relationships. Research to date has not identified an effective way to improve sexual and psychosocial adjustment for both men with prostate cancer and their partners. As well, the efficacy and cost effectiveness of peer counselling as opposed to professional models of service delivery has not yet been empirically tested. This paper presents the design of a three arm randomised controlled trial (peer vs. nurse counselling vs. usual care) that will evaluate the efficacy of two couples-based sexuality interventions (ProsCan for Couples: Peer support vs. nurse counselling) on men's and women's sexual and psychosocial adjustment after surgical treatment for localised prostate cancer; in addition to cost-effectiveness.

**Methods/design:**

Seventy couples per condition (210 couples in total) will be recruited after diagnosis and before treatment through urology private practices and hospital outpatient clinics and randomised to (1) usual care; (2) eight sessions of peer-delivered telephone support with DVD education; and (3) eight sessions of oncology nurse-delivered telephone counselling with DVD education. Two intervention sessions will be delivered before surgery and six over the six months post-surgery. The intervention will utilise a cognitive behavioural approach along with couple relationship education focussed on relationship enhancement and helping the couple to conjointly manage the stresses of cancer diagnosis and treatment. Participants will be assessed at baseline (before surgery) and 3, 6 and 12 months post-surgery. Outcome measures include: sexual adjustment; unmet sexuality supportive care needs; attitudes to sexual help seeking; psychological adjustment; benefit finding and quality of life.

**Discussion:**

The study will provide recommendations about the efficacy of peer support vs. nurse counselling to facilitate better sexual and couple adjustment after prostate cancer as well as recommendations on whether the interventions represent efficient health service delivery.

**Trial Registration:**

ACTRN12608000358347

## Background

### The context of prostate cancer

Prostate cancer is the most common male cancer and second most common cause of cancer death in men in the Western world (excluding non melanoma skin cancer). In Australia, 1 in 11 men will be diagnosed with prostate cancer in their lifetime (0–74 years) and 1 in 82 will die from the disease [1]. In 2003 there were 13,526 Australian men diagnosed with prostate cancer with this number expected to increase to over 18,000 for 2006 [2]. Improved survival from prostate cancer has been demonstrated worldwide [3]. Around half of all newly diagnosed men are predicted to be alive 15 years after diagnosis [3] such that the large cohort of men living with the consequences of diagnosis and treatment is increasing.

The most frequently received treatment for prostate cancer in Australia is radical prostatectomy and the predominance of radical prostatectomy as the primary treatment approach for this cancer is mirrored elsewhere such as in North America [4,5]. While sexual dysfunction after all treatment approaches is common, the trajectory of this dysfunction and severity varies by treatment modality [6]. Men treated with radiation therapy experience less erectile dysfunction (ED) initially following treatment however, in contrast to radical prostatectomy, function is more likely decline over time. In addition, many men now receive neo-adjuvant hormone therapy with radiotherapy, further complicating the course of their sexual adjustment. For radical prostatectomy early adjuvant hormone therapy is uncommon and ED will be immediate due to surgical damage to the neurovascular bundle that lies adjacent to the prostate, with some improvement over the two years after surgery [7]. However, even with nerve sparing surgical techniques that aim to reduce damage to erectile function as few as 18.5% of men report being able to achieve erections firm enough for sexual intercourse two years after surgery [6,7]. Compared with their age mates, men with prostate cancer have a 10 to 15 fold increase in ED [8]. Other distressing effects of treatment include: penile shortening (68% of men), loss of sexual desire (60–80%), less satisfying orgasms (64–87%), overall sexual dissatisfaction (61–91%) [9,10]. These effects can lead to: impaired sexual performance; changes in relationships with women and sexual partners; lost enjoyment of sexual imaginings; decrements in masculine self esteem [10,11]. Problematically, many men are reluctant to seek help for sexual difficulties, with only about half of men seeking medical treatment for ED up to five years after treatment [10]. Reluctance to seek help is particularly problematic for men who receive radical prostatectomy as, for these men, an early return to sexual activity (by three months after surgery) may increase the recovery rate of spontaneous erections and improve responses to ED treatments [12]. Thus, support services for men with prostate cancer that are targeted to sexuality concerns need to reach men who receive radical prostatectomy within weeks of their cancer treatment.

Sexual dysfunction is a shared problem within couples, with regret and loss common among both members of the couple [13]. However, existing medical and support services for men with prostate cancer are oriented towards the patient, do not pay sufficient attention to the couple relationship and virtually ignore the needs of female partners of these men. Partners are more likely to focus on building their husband's self-esteem and putting the sexual dysfunction into perspective within the relationship, and less likely to focus on their own sexual needs [14]. Partners' quality of life is related to their reports of sexual function within the relationship and sexual dysfunction has implications for the longer-term psychosocial well-being of partners [15]. Women often are less focused on finding 'mechanical' treatments to regain erectile function and more open to counseling that might assist the couple to experience intimacy and closeness even if intercourse is not possible [16]. The attention to improving erectile rigidity in the man, for which 'mechanical' treatments are usually needed, may overshadow the partner's needs for sexual pleasure and stimulation [17].

The psychological distress of female partners is increased if they have limited knowledge of what to expect during the course of their husband's treatment and after care, and unmet supportive care needs are often reported. Female partners may be reluctant to share their distress with their husband in order to minimize the stress of the illness on the couple's experience; and may avoid discussing issues that create emotional tension, such as sexual concerns [14]. This lack of communication means that partners often have to deal with their distress and anxiety alone with limited opportunities for psychosocial care [18]. The distress experienced by partners is exacerbated by their husbands' reliance on them for emotional support, with partners having to manage not only their own anxiety, but also the distress of their husbands [14].

Protecting one's partner from emotional distress may have significant costs to one's own well being and diminish relationship quality over time [19]. Patients' and partners' abilities to cope with prostate cancer and subsequent treatment side-effects are interrelated [20] and can negatively impact on the marital relationship [21]. The reactions of partners to sexual dysfunction and the support they provide appears to affect the level of acceptance of sexual changes experienced by men [22]. As well, the female partner's ability to still enjoy sex without major dysfunction is a strong predictor of better sexual satisfaction in the male partner [10]. The disparate needs of couples experiencing sexual dysfunction highlights the need to provide couples with targeted support that promotes communication and adjustment to sexual outcomes. In work with couples in which the woman had breast or gynecological cancer, enhancing couple communication and conjoint coping with cancer treatments significantly enhanced women's sexual satisfaction [23]. Moreover, this couple focused approach increased couple discussion of cancer related issues, and reduced the unhelpful tendency of some people to avoid discussion. In a similar manner, it is proposed that attending to the couple relationship, promoting a sense of conjoint coping and addressing sexual needs within the relationship, will enhance both partners' adjustment to prostate cancer and increase the chance of adherence and better sexual outcomes including erectile function.

### Approaches to Intervention Delivery

By contrast to women, men are less likely to seek help for psychological distress; are under-represented as clients to cancer support services; are reluctant to utilise effective sexual aids after prostate cancer treatment *despite *high levels of dissatisfaction with the sexual outcomes of treatment. Effective support interventions need to utilise delivery methods and sources that are acceptable to this patient group. Men and their partners prefer individual consultations for sexuality support after prostate cancer [16]. Tele-delivered interventions are highly acceptable to this group, and web/computer based programs are frequently accessed by men for medical and procedural information [24,25]. Remote access delivery methods overcome geographical barriers to access and so are applicable to geographically dispersed populations with high potential for population-based translation.

A source of support that has high uptake amongst men with prostate cancer in Australia and internationally is peer support, with men reporting that peer discussions provide informational and emotional support and reduce feelings of social isolation [26]. A feasibility study of a dyadic peer support program for men with prostate cancer reported reduced depression and improved self efficacy in the short term, with men most frequently discussing incontinence, erectile dysfunction and Prostate-Specific Antigen testing with their matched peers [27]. As well, a randomised controlled trial of a group education program to assist men to adjust to prostate cancer treatments [28] found that only by adding peer discussion to the provision of information by an expert was sexual bother alleviated significantly, relative to a control group. An advantage of peer support that is provided by veteran patients is that it is inexpensive by comparison to professionally delivered approaches, such as specialist nurses. While this approach is highly promising, to date randomised controlled trials to assess the effectiveness of peer support in improving men's adjustment have not been undertaken. However, based on research to date a peer delivered counselling intervention paired with education may have equal efficacy to health professional delivery. As well, the relative cost savings for a peer support approach as compared to professional approaches, although not yet quantified, make this a potentially cost effective source of support.

### Intervention Studies Targeting Sexuality

To date intervention research targeting sexuality after prostate cancer is scant. Two trials noted improvements in sexual satisfaction, but not functioning, following general psycho-educational interventions [24,28]. These studies were limited by not including the man's partner [24,28]; not targeting men early in the cancer treatment continuum [28]; and not controlling for type of cancer treatment [24,28]. One of the only intervention studies to focus specifically on improving sexual function was a randomized trial comparing four face-to-face couple counselling sessions to similar sessions for the man alone, with the female partner just reading educational material and collaborating with homework tasks [17]. Men and their partners in both conditions reported improved sexual function and satisfaction at three month follow up and increased utilisation of medical treatments for ED at three and six months; gains in sexual function diminished at six months. Study limitations included low statistical power from a small sample size and that as men were an average of 27 to 30 months post-treatment at baseline, the critical opportunity for early intervention was missed. As well, face-to-face delivery method is relatively expensive, hard to access, and difficult to translate into a population-based cost-effective approach.

We propose that greater attention to the couple relationship in the intervention would improve female sexual or couple relationship satisfaction. Moreover, given the strong association between sexual and relationship satisfaction, particularly for women [29], enhancing the couple relationship is likely to improve long-term maintenance of sexual satisfaction improvements.

## Methods/Design

### Study Aims and Hypotheses

The overall study aim is to compare the efficacy of peer-delivered telephone support with DVD educational resource, vs. oncology nurse-delivered telephone counselling with DVD educational resource, vs. usual care in improving both men's and women's sexual and psychosocial adjustment at 3, 6 and 12 months after treatment for localised prostate cancer. In doing so we will also compare the cost-effectiveness of support by trained peers vs. nurse counsellors; and identify demographic, medical and psychosocial variables that predict improvement in psychosexual adjustment in prostate cancer patients and their partners with each intervention approach.

The intervention will utilise a cognitive behavioural approach that has been found to be effective in promoting positive adjustment after cancer [30], along with couple relationship education focussed on relationship enhancement and helping the couple to conjointly manage the stresses of cancer diagnosis and treatment [19]. The study will have three arms: (1) usual care (2) telephone support by a trained male peer support volunteer who is a prostate cancer survivor with DVD education and (3) oncology nurse-delivered telephone counselling with DVD education.

It is hypothesised that 3, 6, and 12 months after surgery for localised prostate cancer:

1. By contrast to couples in usual care, couples who receive either the peer or nurse delivered intervention will have a more positive sexual adjustment; lower unmet sexuality supportive care needs; more positive attitudes to sexual help seeking; higher uptake of erectile aids; improved psychological adjustment and quality of life.

2. Couples who receive the peer and nurse delivered intervention will have similar sexual adjustment; sexuality supportive care needs; attitudes to sexual help seeking; uptake of erectile aids; psychological adjustment and quality of life.

3. The peer delivered intervention will be more cost effective by comparison to the nurse delivered intervention.

### Intervention

Usual care will consist of the man's standard medical management and existing written educational materials. For the two intervention arms, the eight sessions of phone support/counselling will include enhanced couple communication and conjoint coping content and material relevant to the early treatment phase. An audiovisual DVD resource with Tip Sheets will accompany the intervention to enhance the psycho-education and sexuality education components and to also provide actor role models for effective couple communication about sexuality and intimacy. The nurse counselling sessions will follow principles of cognitive-behavioural sex and marital therapy and will utilise an adult learning approach in which partners' self-select goals to focus on while working through the program. Content includes education about prostate cancer, menopause, and sexuality; assigned behavioural homework including increasing expression of affection and non-demanding sexual touch; challenging negative beliefs about prostate cancer, aging, and sexuality; and helping the couple choose a medical treatment for ED that is acceptable to both partners, and integrating this into their sexual relationship. Additional components that target the challenges of the early treatment phase (e.g., urinary incontinence, pain, sleep disturbance, psychological distress) will be additionally selected by the couple if relevant.

Peer support is based on the support partner or 'veteran' patient having personal experience and knowledge about the cancer experience; a unique personal insight into effective ways to cope; and the ability to form a support relationship that is derived from the connection of shared experience. In this way peer support can reduce feelings of isolation and stigma (the sense of being the 'only one'); can convey emotional, social, informational and practical support; and through role modelling can communicate realistic hope and optimism about the future. Peer support volunteers will be prostate cancer survivors who are at least 12 months post treatment and who have support group experience. The intervention will follow the same couples-based approach as the nurse counselling intervention but will be oriented to empathic mutual support and education rather than in depth sex and marital therapy.

The patient's partner will be invited to participate in all phone sessions, and actual participation will be recorded by the peer/therapist for each phone session, as well as minutes of counselling time, for inclusion in analyses. Support/counselling calls are timed to correspond with the challenges associated with preparing for and recovering from radical prostatectomy. The first two calls will occur prior to surgery; four fortnightly calls (Sessions 3 to 6) are timed to commence two weeks after surgery; a further two calls (Session 7 to 8) 16 and 22 weeks post-surgery.

### Participants

With the strong endorsement and support of Queensland Urologists, patients will be referred to the project from private urology practices and public hospital outpatient clinics in Queensland, Australia. Informed written consent will be obtained by study trained research nurses who will contact potential participants after referral to the study. We will recruit 70 couples per condition over a 12 month period (allowing for 10% attrition from treatment; 210 couples in total to be recruited). Assuming a moderate effect size of d = 0.5, alpha at .05, the resulting power with 70 couples per condition is 0.8. Inclusion criteria are that the men must: (1) have been newly diagnosed with localised prostate cancer and have chosen radical prostatectomy as their treatment approach (2) be currently in a heterosexual cohabitating couple relationship (3) be able to read and speak English (4) have no previous history of head injury, dementia or psychiatric illness (5) have no other concurrent cancer. As the intervention has been developed based on previous data for heterosexual couples this intervention is unlikely to be helpful for homosexual couples.

### Study Integrity

Ethical approval has been obtained from the Griffith University Human Research Ethics Committee. The study design will be guided by the CONSORT statement [31]. Randomisation to study condition will occur following the completion of baseline assessment. Assessments will be by self-report pen and paper measures and project staff tracking assessments will be blinded to condition. Randomisation will occur in blocks of 12, with each condition randomly generated 4 times within each block to ensure an unpredictable allocation sequence with equal numbers of couples in each group at the completion of each block. This sequence will be undertaken by the project manager and concealed from investigators. Therapy will be manualised and all intervention calls audiotaped with 25% reviewed to ensure treatment adherence. All analyses will be conducted on the basis of intention to treat.

### Measures

A series of previously validated and reliable self report measures will be administered by mail. Domain specific quality of life (QOL) will be included as a potential moderator of intervention effect and challenge appraisals and therapeutic alliance as mediators. Primary outcomes are: sexual adjustment; unmet sexual supportive care needs; masculine self-esteem; marital satisfaction; utilisation of erectile aids. Secondary outcomes are: psychological distress; overall QOL and benefit finding. Disease variables (e.g. cancer grade, stage) will be assessed through medical and cancer registry records review. Use of medical services and associated costs will be assessed through Medicare Australia records.

#### Moderators/Mediators

##### Domain specific QOL

The International Prostate Symptom Score [32] and the urinary and bowel symptom subscales of the UCLA Prostate Cancer Index [33] will assess disease specific QOL. Women will complete a menopausal symptom scale derived from the Breast Cancer Prevention Trial (BCPT) Symptom Checklist [34].

##### Challenge appraisal

A person's cognitive appraisal of an event will determine if that event is perceived as stressful and this will be assessed using a Stress Appraisal Measure based on the work of Roesch [35].

##### Therapeutic alliance

The quality of the bond between the peer and nurse counsellors and the couple and extent of agreement about therapy goals will be assessed by the Working Alliance Inventory [36].

#### Primary Outcome Variables

##### Sexual function

Men will complete the International Index of Erectile Functioning (IIEF) [37], which allows sexual function to be assessed in five domains: erectile function, orgasmic function, sexual desire, intercourse satisfaction and overall sexual satisfaction. Women will complete the Female Sexual Function Index (FSFI) [38]. This questionnaire parallels the IIEF and examines sexual function among women in six domains: sexual desire, arousal, lubrication, orgasm, satisfaction, and pain.

##### Sexual Supportive Care Needs

Couples needs related to sexual relationships will be assessed using the sexuality needs subscale of the Supportive Care Needs Survey [39,40].

##### Sexual Self-Confidence

The Short Form Psychological and Interpersonal Relationship Scale (SF-PAIRS) [41] will assess sexual confidence and spontaneity associated with ED.

##### Masculine Self-Esteem

The Masculine Self-Esteem scale will assess men's appraisal of their masculinity [42].

##### Utilisation of sexual aids

A scale developed by Schover [43] will assess whether men have obtained medical help for sexual dysfunction and the impact of each treatment on their sex life.

##### Marital satisfaction

An abbreviated version of the Dyadic Adjustment Scale (A-DAS) [44] will assess marital satisfaction among couples.

#### Secondary Outcome Variables

##### Psychological Distress

The Hospital Anxiety and Depression Scale [45] will provide a global measure of current psychological distress with subscale scores for anxiety and depression.

##### Quality of Life

Health related quality of life will be assessed with the SF-36, the most widely used QOL measure in the world with norms for the Australian general population available. The SF-36 [46] contains a mental health and physical health summary scale to measure the impact of the intervention on patients' and partners' wellbeing.

##### Benefit Finding

Benefit finding [47] will be used to measure the perceived positive experiences and outcomes (eg appreciation of life, changes in life priorities) resulting from the diagnosis of cancer.

### Statistical Analyses

The study hypotheses will be tested by multilevel modelling (MLM). This class of procedures is the appropriate way to analyse hierarchical data sets such as the longitudinal data of the proposed research in which observations are nested within persons who in turn are nested within couples. Study condition is modelled as a fixed effect at the couple level. The typical RCT effects that have been tested by ANOVAs are all available with MLM; however there are several fundamental differences between the statistical models. First, in MLM individual and couple trajectories of change in time can be modelled directly as random effects. This provides appropriate tests of Hypotheses 1 and 2 and allows precise examination of predictors of individual versus group change. Second, MLM minimises the loss of data through attrition in that unlike ANOVA all available data points from participants are included in analyses. The direct ML estimation normally used in MLM is currently the most favoured technique (along with multiple imputation) for minimising bias and enhancing precision in parameter estimates from incomplete data [48]. Hypotheses 1 and 2 will be tested with appropriate contrasts on the fixed effect of study condition. Although power calculations from multilevel longitudinal analyses are not as well articulated as for older techniques, the study will have at least as much power as the equivalent ANOVA (i.e., 80% for a moderate effect size;) as the intervention effects are all based upon the level 3 (i.e., couple) sample size.

A cost-utility analysis will also be undertaken to address Hypothesis 3 where intervention resources and health outcomes are combined in an analysis to produce information on the relative economic efficiency between the peer, nurse specialist and usual care options. The analysis will take the perspectives of the health provider and health system and involve the assessment of 1) cost data on resources used in each of the three arms by identifying, quantifying and valuing resources using standard methods and, 2) health outcomes, in terms of quality-adjusted life years (QALYs). Using Medicare Australia data, we also wish to capture health utilisation costs for GP visits and medication use to assess whether the interventions change typical health care use. Quality of life will be measured in participants using the preference-based utility instrument SF-6D [49] which is based on the SF-36 quality of life tool. The key outcome for the cost-utility analysis will be the *incremental cost-effectiveness ratio*, expressed as the incremental cost per QALY. This ratio represents the difference in costs between the intervention and usual care options divided by the difference in QALYs gained across the two options. This means that it is the *additional *cost and health benefits of each of the two interventions over and above what occurs in usual care that is important. Secondary economic endpoints will include incremental cost-effectiveness ratios for cost per % gain in sexual function and psychological distress. A Bayesian statistical approach to the analysis will be followed so that probabilistic statements on the efficiency of the intervention will be produced [50]. Data will be analysed using the computer program TreeAge Pro (Healthcare Module) 2005 [51]. The results will be scrutinised using probabilistic sensitivity analysis which is standard practice in economic evaluations to address data uncertainty and potentially strengthens the generalisability of the results. Specifically, Monte Carlo simulations will produce cost-effectiveness acceptability curves and probabilistic statements on cost-effectiveness.

## Discussion

This study will address a critical but as yet unanswered research question: to identify a cost-effective and population based approach to promoting optimal psychosexual adjustment for men with prostate cancer and their partners. To date, for this patient group, no sexuality intervention studies have: targeted couples at diagnosis when distress is highest; been adequately powered to look differentially at intervention effects; trialled peer support; or included economic analyses. This research will overcome these limitations. The intervention will be able to be utilised by trained nurses in a range of settings including broad reach tele-health lines and also through peer support programs that are conducted internationally. This means that project outputs will be immediately translatable into practice to improve the sexual health and overall well-being of men with prostate cancer and their partners.

## Competing interests

The authors declare that they have no competing interests.

## Authors' contributions

SKC and JD developed the study concept and aims and initiated the project. LS, KH, MF, LG, RAG and SO assisted in further development of the protocol. SKC was responsible for drafting the manuscript. SKC, SC, MF, SO and JD will implement the protocol and oversee collection of the data. All authors contributed to the final manuscript.

## Pre-publication history

The pre-publication history for this paper can be accessed here:


